# Brain Motor Network Changes in Parkinson's Disease: Evidence from Meta‐Analytic Modeling

**DOI:** 10.1002/mds.28468

**Published:** 2021-01-11

**Authors:** Damian M. Herz, David Meder, Julia A. Camilleri, Simon B. Eickhoff, Hartwig R. Siebner

**Affiliations:** ^1^ Danish Research Centre for Magnetic Resonance, Centre for Functional and Diagnostic Imaging and Research Copenhagen University Hospital Hvidovre Hvidovre Denmark; ^2^ Research Center Juelich Institute of Neuroscience and Medicine, Brain & Behaviour (INM‐7) Juelich Germany; ^3^ Institute of Systems Neuroscience, Medical Faculty Heinrich Heine University Duesseldorf Duesseldorf Germany; ^4^ Department of Neurology Copenhagen University Hospital Bispebjerg Copenhagen Denmark; ^5^ Institute of Clinical Medicine, Faculty of Health and Medical Sciences University of Copenhagen Copenhagen Denmark

**Keywords:** Parkinson's disease, functional neuroimaging, meta‐analysis, motor

## Abstract

**Background:**

Motor‐related brain activity in Parkinson's disease has been investigated in a multitude of functional neuroimaging studies, which often yielded apparently conflicting results. Our previous meta‐analysis did not resolve inconsistencies regarding cortical activation differences in Parkinson's disease, which might be related to the limited number of studies that could be included. Therefore, we conducted a revised meta‐analysis including a larger number of studies. The objectives of this study were to elucidate brain areas that consistently show abnormal motor‐related activation in Parkinson's disease and to reveal their functional connectivity profiles using meta‐analytic approaches.

**Methods:**

We applied a quantitative meta‐analysis of functional neuroimaging studies testing limb movements in Parkinson's disease comprising data from 39 studies, of which 15 studies (285 of 571 individual patients) were published after the previous meta‐analysis. We also conducted meta‐analytic connectivity modeling to elucidate the connectivity profiles of areas showing abnormal activation.

**Results:**

We found consistent motor‐related underactivation of bilateral posterior putamen and cerebellum in Parkinson's disease. Primary motor cortex and the supplementary motor area also showed deficient activation, whereas cortical regions localized directly anterior to these areas expressed overactivation. Connectivity modeling revealed that areas showing decreased activation shared a common pathway through the posterior putamen, whereas areas showing increased activation were connected to the anterior putamen.

**Conclusions:**

Despite conflicting results in individual neuroimaging studies, this revised meta‐analytic approach identified consistent patterns of abnormal motor‐related activation in Parkinson's disease. The distinct patterns of decreased and increased activity might be determined by their connectivity with different subregions of the putamen. © 2021 The Authors. *Movement Disorders* published by Wiley Periodicals LLC on behalf of International Parkinson and Movement Disorder Society.

Parkinson's disease (PD) is a common and disabling neurodegenerative disorder. Even though many patients develop nonmotor symptoms, such as depression or autonomic dysfunction, the disease is still considered a movement disorder and is defined by the hallmark presence of bradykinesia, that is, the slowing of movement initiation and progressive reduction in speed and amplitude of repetitive movements.^1^, ^2^ Bradykinesia can be conceptualized as an impaired ability to “energize” or “charge” movements and has been attributed to an impaired modulation of movement vigor.^3^, ^4^ To better understand the neural underpinning of this motor impairment, a multitude of studies have been conducted using neuroimaging techniques, such as functional magnetic resonance imaging (fMRI) and H_2_O^15^ positron emission tomography (PET) while patients perform a motor task. However, the results of these studies often seem conflicting. For example, several studies reported decreased activity in the medial prefrontal and frontal cortices in PD,^5^, ^6^, ^7^, ^8^ whereas other studies reported activity in these areas to be increased.^9^, ^10^, ^11^, ^12^ One approach to addressing these inconsistencies is to conduct meta‐analyses to overcome some of the shortcomings of neuroimaging studies in PD, such as small sample size and heterogeneity of the studied patient group. Furthermore, it allows the generalization of findings beyond the precise experimental setup and task design of a specific study. Thus, meta‐analyses allow assessing whether there are differences in neural activation in PD that are consistent across individual patient groups and motor tasks. We previously conducted a meta‐analysis of neuroimaging studies in PD^13^ using a quantitative, coordinate‐based approach termed activation likelihood estimation (ALE). This analysis pinpointed the motor territory of the striatum, the posterior putamen, as the brain region that was most consistently underactivated during motor tasks in PD. At the cortical level, the observed frontal and parietal activation differences were less consistent regarding the directionality of changes (ie, increased or decreased in PD relative to healthy controls) and appeared to rely more strongly on the applied motor task. This raises the question whether cortical activation changes in PD are task dependent rather than reflecting general disease‐related neural dysfunction. An alternative explanation for the discordant results from our previous meta‐analysis is the limited number of studies that could be included at the time, because meta‐analyses with a small number of included studies have relatively low statistical power and can be strongly affected by results from individual experiments.^14^ To address this, we conducted a revised ALE meta‐analysis, which included an additional 15 studies, reporting data from an additional 285 patients that were published after our previous meta‐analysis. Furthermore, we computed functional connectivity profiles of the abnormally activated areas to further characterize the dysfunctional motor networks underlying PD.

## Methods

### Literature Search and Study Selection

We conducted a search on PubMed using the identical search strings as in our previous meta‐analysis:^13^ (“Parkinson's disease” OR “Parkinson disease” OR “Parkinsons disease”) AND (“functional magnetic resonance” OR “fMRI” OR “positron emission tomography” OR “PET”). The final search was conducted on June 30, 2020, and resulted in 3841 studies. We did not find any additional articles through review articles and reference tracing. We only screened studies using fMRI or H_2_O^15^‐PET during motor paradigms that were written in the English language, resulting in 170 studies that were further assessed by reading the abstract and/or main text. The following exclusion criteria were then applied for all experiments:

1. Review articles reporting no original data or PET studies other than H_2_O^15^‐PET (n = 20).
2. Studies testing passive movements, eye movements (saccades), speech, motor learning, or executive control, for example, task switching (n = 28).
3. Motor tasks were tested against each other rather than against baseline or a nonmotor control task, for example, fixation (n = 19).
4. Neither of the contrasts “PD OFF medication versus healthy controls,”, “PD ON medication versus healthy controls,” or “PD ON medication versus PD OFF medication” were statistically compared (n = 19).
5. Analyses were based on regions of interest (n = 29). These most commonly comprised the putamen and other basal ganglia areas, primary motor cortex, supplementary motor areas, cerebellum, and, less frequently, parietal or other cortical areas. Some studies in particular early publications did not cover the whole brain. These studies, however, were not excluded because they did not include regions based on a priori assumptions, and in many studies the field of view was not reported. Likewise we did not exclude studies that masked the between‐group comparisons based on task‐related activity in the control group because this was not based on a priori assumptions about the brain areas of interest.
6. Multivariate analyses or covariance analyses (n = 6).
7. Fewer than 6 PD patients were included (n = 2).
8. Studies in which PD patients were treated with deep brain stimulation or received acute challenges with drugs other than levodopa (eg, apomorphine), because these treatments induce distinct effects on the sensorimotor system in PD^15^, ^16^ (n = 5).

As in our previous meta‐analysis, another study^17^ was excluded because of a significant age difference between the PD and control groups. If coordinates were not reported, we contacted the corresponding author by email (coordinates could not be obtained in 3 studies). This procedure resulted in the exclusion of 131 studies, leaving 39 studies that were included.^5^, ^6^, ^8^, ^9^, ^10^, ^11^, ^12^, ^18^, ^19^, ^20^, ^21^, ^22^, ^23^, ^24^, ^25^, ^26^, ^27^, ^28^, ^29^, ^30^, ^31^, ^32^, ^33^, ^34^, ^35^, ^36^, ^37^, ^38^, ^39^, ^40^, ^41^, ^42^, ^43^, ^44^, ^45^, ^46^, ^47^, ^48^, ^49^ Fifteen of these studies were published after our previous meta‐analysis and allowed us to conduct a well‐powered meta‐analysis. For an overview of the included studies, please see Table 1.

**TABLE 1:** Studies included in the meta‐analysis

Study	Modality	PD, n	C, n	UPDRS‐III OFF	UPDRS‐III ON	Age of PD	Age of C	Foci, n	Contrast
Baglio et al, 2011	fMRI	15	11		21.5	66.5	66.9	6	HC vs ON
Task:	Button press with right index finger								
Buhmann et al, 2003	fMRI	8	n/a			54	n/a	2	ON vs OFF
Task:	Random finger opposition task at 0.33 Hz with right and left hands								
Burciu et al, 2015	fMRI	20	20	31.9		65.8	64.8	20	HC vs OFF
Task:	Grip force task with or without feedback with more affected hand								
Caproni et al, 2013	fMRI	11	11	20		65	65.1	7	HC vs OFF
Task:	Tapping with right index finger								
	fMRI	11	11	20		65	65.1	15	HC vs OFF
Task:	Sequence from dig I to V with right hand								
	fMRI	11	11	20		65	65.1	13	HC vs OFF
Task:	Sequence with order dig I, III, V, II, IV with right hand								
Cerasa et al, 2006	fMRI	10	11	27.5		64.2	63.4	8	HC vs OFF
Task:	Synchronized tapping with right index finger at 1.33 Hz								
	fMRI	10	11	27.5		64.2	63.4	3	HC vs OFF
Task:	Continuation of the tapping with right index finger without stimulus								
Drucker et al, 2019	fMRI	22	19	33.9		67.7	64.7	4	HC vs OFF
Task:	Externally cued foot‐tapping sequence								
	fMRI	22	19	33.9		67.7	64.7	6	HC vs OFF
Task:	Internally cued foot‐tapping sequence								
Eckert et al, 2006	fMRI	9	9	20.6	10.7	63.3	60.6	18	HC vs OFF
	fMRI	9	9	20.6	10.7	63.3	60.6	9	HC vs ON
	fMRI	9	n/a	20.6	10.7	63.3	n/a	4	ON vs OFF
Task:	Opening and closing of right fist at ~1 Hz								
Gonzalez‐Garcia et al, 2011	fMRI	17	10		41	64.4		8	HC vs ON
Task:	Button presses with right and left hands in predefined order								
	fMRI	17	10		41	64.4		5	HC vs ON
Task:	Button presses with right and left hands in random order								
Haslinger et al, 2001	fMRI	8	8	15.8	11.8	60.8	54.4	7	HC vs OFF
	fMRI	8	8	15.8	11.8	60.8	54.4	8	HC vs ON
	fMRI	8	n/a	15.8	11.8	60.8	n/a	10	ON vs OFF
Task:	Joystick movements with right hand with 4 spatial degrees of freddom (dof)								
Holiga et al, 2012	fMRI	12	n/a	33.5	9.6	56	n/a	5	ON vs OFF
Task:	Index‐to‐thumb opposition movements with right and left hands at 1 Hz								
Hughes et al, 2010	fMRI	16	15	31.3	18.9	63.9	66.5	10	HC vs ON
Task:	Specified and chosen button presses with right hand								
Jia et al, 2018	fMRI	22	22	16.45		61	60.6	8	HC vs OFF
Task:	Self‐initiated tapping with right index finger at approximately 0.5 Hz								
Katschnig et al, 2011	fMRI	20	20	37.9		66.8	62.3	2	HC vs OFF
Task:	Dorsiflexion of right and left ankles at 1 Hz								
Kim et al, 2018	fMRI	16	15	36		63.1	64.1	6	HC vs OFF
	fMRI	16	15	36		63.1	64.1	19	HC vs ON
	fMRI	16	n/a	36		63.1	n/a	5	ON vs OFF
Task:	Two‐choice forced response task with fingers II and III of right hand								
Kraft et al, 2009	fMRI	12	12	21	13.9	60.8	53	12	HC vs OFF
	fMRI	12	12	21	13.9	60.8	53	8	HC vs ON
	fMRI	12	n/a	21	13.9	60.8	n/a	4	ON vs OFF
Task:	Grip‐force task with right and left hands simultaneously								
	fMRI	12	12	21	13.9	60.8	53	13	HC vs OFF
	fMRI	12	12	21	13.9	60.8	53	4	HC vs ON
	fMRI	12	n/a	21	13.9	60.8	n/a	4	ON vs OFF
Task:	Grip‐force task with right and left hands alternating								
Maillet et al, 2012	fMRI	12	n/a	40.3	10	59.8	n/a	2	ON vs OFF
Task:	Joystick movements with right hand with 4 spatial dof at 0.5 Hz								
Mak et al, 2016	fMRI	26	21		29	61.4	60.9	3	HC vs ON
Task:	Self‐initiated index finger tapping at ~0.2–0.3 Hz on most affected side								
	fMRI	26	21		29	61.4	60.9	3	HC vs ON
Task:	Cued index finger tapping at ~0.2–0.3 Hz on most affected side								
Mallol et al, 2007	fMRI	13	11	22.6		64.9	61.9	13	HC vs OFF
Task:	Finger‐to‐thumb opposition and rotating movements of right hand								
Martin et al, 2019	fMRI	22	22	15.6		53	48.5	13	HC vs OFF
Task:	Self‐generated sequential button press with fingers I‐IV of most affected hand								
	fMRI	22	22	15.6		53	48.5	10	HC vs OFF
Task:	Self‐generated sequential button press with fingers I‐IV of less affected hand								
	fMRI	22	22	15.6		53	48.5	13	HC vs OFF
Task:	Visually‐cued sequential button press with fingers I‐IV of most affected hand								
	fMRI	22	22	15.6		53	48.5	5	HC vs OFF
Task:	Visually‐cued sequential button press with fingers I‐IV of less affected hand								
Mattay et al. 2002	fMRI	7	n/a	8.8	5	55	n/a	7	ON vs OFF
Task:	Button presses with right hand (0‐back task)								
Mohl et al, 2017	fMRI	26	21	33	24	62.2	61.6	1	HC vs OFF
Task:	1 Hz sequential tapping from fingers I‐V and vice versa with right hand								
Payoux et al, 2011	PET	8	10	22	12	62	67	3	HC vs OFF
	PET	8	n/a	22	12	62	n/a	1	ON vs OFF
Task:	Joystick movements with right hand with 4 spatial dof at 0.33 Hz								
Pinto et al, 2011	fMRI	9	15	33		59	55	6	HC vs OFF
Task:	Joystick movements with right hand with 4 spatial dof at 0.5 Hz								
Planetta et al, 2015	fMRI	14	14	29.6		64	61.9	34	HC vs OFF
Task:	Cued and memorized pinch grip force task with most affected hand (collapsed)								
Poisson et al, 2013	fMRI	6	10	16		65	53.6	13	HC vs OFF
Task:	Finger‐thumb tapping with right hand at 1 Hz								
Rottschy et al, 2013	fMRI	23	23		23.9	67.2	65	8	HC vs ON
Task:	Direct repeat of sequence of 4 or 5 finger movements with both hands								
	fMRI	23	23		23.9	67.2	65	14	HC vs ON
Task:	Delayed repeat of sequence of 4 or 5 finger movements with both hands								
Rowe et al. 2002	fMRI	12	12	33.7		62	62	2	HC vs OFF
Task:	Sequential finger movements of right hand at 0.33 Hz								
Sabatini et al, 2000	fMRI	6	6	16		61	59	15	HC vs OFF
Task:	Finger‐to‐thumb opposition movements and fist clenching with right hand								
Samuel et al, 1997	PET	6	6	17.7		70.2	64.3	7	HC vs OFF
Task:	Sequential finger movements of right hand at 0.33 Hz								
	PET	6	6	17.7		70.2	64.3	10	HC vs OFF
Task:	Bimanual sequential finger movements at 0.33 Hz								
Tessa et al, 2010	fMRI	15	11	16.1		70.1	69	12	HC vs OFF
Task:	Continuous tapping of right hand								
Tessa et al, 2012	fMRI	15	13	16.3		68.1	64.2	4	HC vs OFF
Task:	Continuous writing of the figure “8” with right hand								
Tessa et al, 2013	fMRI	11	10	13.5		67.7	64	6	HC vs OFF
Task:	Continuous tapping of left hand								
Turner et al, 2003	PET	12	12	41.4		57	58	9	HC vs OFF
Task:	Tracking task with right hand								
Wu et al, 2005	fMRI	12	12	25.5		61.2	61.8	12	HC vs OFF
Task:	Sequential finger tapping with right hand at ~0.5 Hz								
Wu et al, 2010	fMRI	15	15	20.7		59.7	60.3	15	HC vs OFF
Task:	In‐phase movements of both index fingers at ~0.5 Hz								
	fMRI	15	15	20.7		59.7	60.3	20	HC vs OFF
Task:	Antiphase movements of both index fingers at ~0.5 Hz								
Wu et al, 2015	fMRI	26	26	13		59	58.9	7	HC vs OFF
Task:	Tapping with right index finger at 0.3–0.5 Hz								
Wu et al, 2016	fMRI	18	18	20.4		60.4	59.9	11	HC vs OFF
	fMRI	18	n/a	20.4		60.4	n/a	7	ON vs OFF
Task:	Free writing in PD patients with consistent micrographia								
	fMRI	18	18	19.1		59.6	60	9	HC vs OFF
	fMRI	18	n/a	19.1		59.6	n/a	4	ON vs OFF
Task:	Free writing in PD patients with progressive micrographia								
Wurster et al, 2015	fMRI	10	10		20.7	66.4	64.9	2	HC vs ON
Task:	Auditory‐cued button press with right index finger at 1, 2.5, and 4 Hz (collapsed)								
Yan et al, 2015	fMRI	11	12	20.1		61.5	65.5	5	HC vs OFF
Task:	Auditory‐cued finger‐to‐thumb movement with left hand								
	fMRI	11	12	20.1		61.5	65.5	4	HC vs OFF
Task:	Auditory‐cued finger‐to‐thumb movement with right hand								

HC, healthy control participants; OFF, Parkinson's disease patients off dopaminergic medication; ON, Parkinson's disease patients on dopaminergic medication; foci, number of activation foci reported in the respective study; n/a, not applicable.

### Activation Likelihood Estimation Meta‐Analysis

The meta‐analyses were carried out using the revised version^50^ of the activation likelihood estimation approach for coordinate‐based meta‐analyses.^51^ Activation likelihood estimation (ALE) tests whether there is a significant convergence between activation foci from different experiments compared with a random distribution of foci. Because the term “experiment” refers to a contrast of interest (eg, PD‐ON vs PD‐OFF) for a given study, 1 study can contribute with several experiments to the ALE. A detailed description of the ALE technique can be found elsewhere.^50^, ^52^ In short, activation foci from different experiments were modeled as spatial 3‐dimensional Gaussian probability distributions, where the size of the distribution depends on the number of participants in the respective experiment (in case of different numbers of participants for the PD and healthy control groups, the lower number was used). If coordinates were reported in Talairach space, they were transformed to Montreal Neurological Institute space using the tal2icbm method.^53^ Combining probabilities for foci in each experiment resulted in a modeled activation (MA) map. Subsequently, voxel‐wise ALE scores were computed by taking the union of the MA maps describing the convergence of results across experiments at each gray matter voxel. The nonparametric *P* values of ALE scores were derived by the proportion of equal or higher values obtained under the assumption of random spatial association and thresholded at a cluster level–corrected threshold of *P* < 0.05 family‐wise error–corrected.

Since publication of our first meta‐analysis,^13^ it has been demonstrated that the results of meta‐analyses comprising only few experiments are driven by single studies. We therefore now only conducted meta‐analyses for contrasts based on >20 experiments.^14^ Thus, no meta‐analyses were conducted for the contrasts PD‐ON versus healthy controls (13 experiments for healthy controls (HC) > PD‐ON and 7 experiments PD‐ON > HC) or PD‐ON versus PD‐OFF (10 experiments for PD‐ON > PD‐OFF and 5 experiments for PD‐OFF > PD‐ON). For the same reason we did not conduct analyses separately for motor tasks that were externally or internally cued (there were <20 experiments for all contrasts with internally timed and internally chosen movements). There were sufficient experiments to conduct meta‐analyses for the contrasts PD‐OFF > HC (34 experiments) and HC > PD‐OFF (36 experiments). We also conducted meta‐analyses comparing HCs and PD patients irrespective of medication (ie, irrespective of whether patients were ON or OFF medication), which included 41 experiments for the contrast PD > HC and 49 experiments for the contrast HC > PD.

Even though there is currently no optimal approach to conduct ALE correlation analyses across the whole brain, we attempted to relate the observed underactivation in PD to disease severity as indexed by the mean UPDRS scores of the individual studies. To this end, we computed how much individual studies contributed to a given cluster and then entered this variable into a nonparametric Spearman correlation with the mean UPDRS score.

We also computed the probability of experiments detecting abnormal activation of the putamen in PD. To this end, we assessed whether a given experiment activated the putamen in the control group (detected in 25 experiments) and whether this experiment found decreased putamen activity in PD. This additional analysis was motivated by the observation that in many experiments the motor task mainly induced activation in cortical areas and less frequently in the basal ganglia. Given the important role of the putamen in motor symptoms in PD,^2^ this lack of striatal engagement seemed surprising and might be because of the specific experimental design and data acquisition. This analysis thus tried to circumvent this problem by only looking at the subsample of studies revealing putamen activation in healthy participants. We could not perform the same analysis for cortical and cerebellar changes because the exact localization of activation in healthy controls was often not given and we could not distinguish between activation of, for example, pre‐SMA versus SMA or rostral premotor versus precentral gyrus. On the other hand, in the case of basal ganglia activation, it was explicitly mentioned whether the putamen was activated in almost all studies.

### Meta‐Analytic Connectivity Modeling

After having established which foci showed consistent differences in activation between PD patients and healthy controls, we further analyzed these foci regarding their functional task‐related connectivity profiles. Meta‐analytic connectivity modeling (MACM) tests consistent coactivation patterns of a volume of interest (VOI) with the rest of the brain. In short, experiments in healthy subjects, which report activation at the VOI (here, the foci with consistent activation differences from the ALE analysis) were retrieved from the BrainMap database.^54^, ^55^ A coordinate‐based meta‐analysis was then performed using ALE, which generates a coactivation pattern across the whole brain for each voxel in each VOI. In other words, the computed pattern reflects which brain areas a given region is commonly coactivated with in healthy subjects, reflecting its functional task‐related connectivity profile. For more details, see reference 56. Because dopaminergic deafferentation of the putamen in PD shows a rostrocaudal gradient with the most pronounced deafferentation in the caudal (posterior) putamen and relatively preserved innervation of the rostral (anterior) putamen, we hypothesized that activity of cortical areas that are primarily connected with the posterior putamen might be more affected in PD compared with cortical areas that are connected to more anterior parts of the putamen. To test this, we analyzed where the coactivation patterns of the different VOIs overlapped, indicating common functional connectivity. To minimize lateralization (eg, left M1 is primarily connected with left putamen), we only used cortical VOIs from the hemisphere contralateral to the most frequently used right hand (only 5 and 6 experiments, respectively, used the left hand for the contrasts HC > PD and PD > HC) in case of bilateral VOIs. Thus, based on the results from the ALE analysis (see below), we used left M1, SMA, and right cerebellum as VOIs for the contrast HC > PD and left rostral precentral gyrus/middle frontal gyrus and pre‐SMA as VOIs for the contrast PD > HC. We then computed 2 overlap images, one of the MACM maps of each of the VOIs for the contrast HC > PD and one for the VOIs for the contrast PD > HC. These 2 overlap images reflect which functional connectivity patterns are common for all VOIs of each contrast. Because we were mostly interested in the putamen (see above), we then used a mask of the bilateral putamen created using the automated anatomical labeling atlas^57^ to assess which areas of the putamen were consistently coactivated with areas that were more and less activated in PD.

## Results

Thirty‐nine publications (36 fMRI, 3 H_2_O^15^‐PET) were included. Meta‐analyses were conducted for contrasts comparing HCs with PD patients irrespective of medication as well as contrasts comparing HCs with PD patients OFF medication. Because only 3 studies used H_2_O^15^‐PET, we also conducted the same meta‐analyses without including H_2_O^15^‐PET studies, which yielded identical results. The number of experiments was too low for comparing HCs with PD patients ON medication or comparing PD patients ON versus OFF medication (see Methods for more details).

### Decreased Activation in Patients with PD

**FIG. 1:**
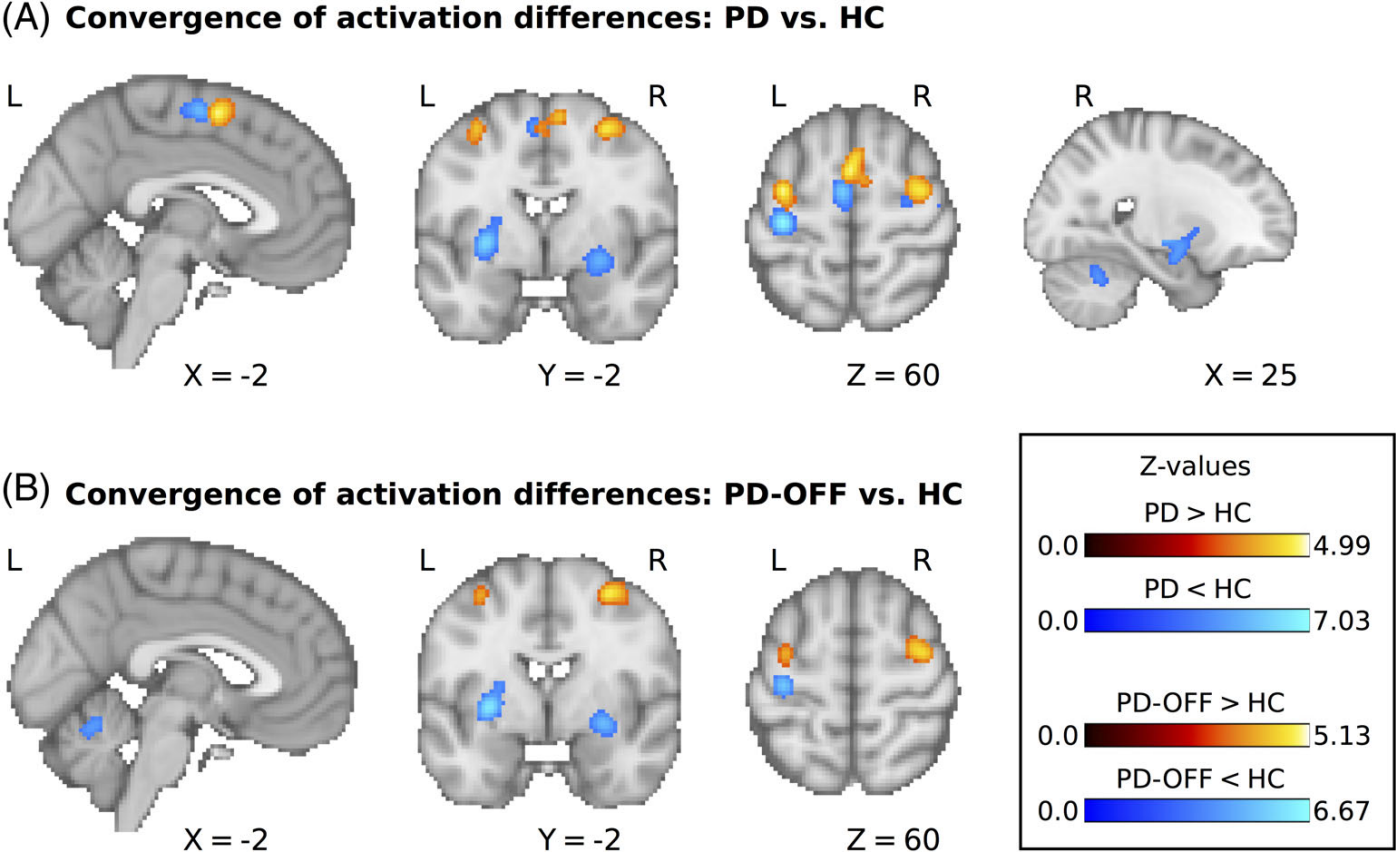
(**A**) Significant clusters for the comparison of motor‐related activity between PD patients and HC. (**B**) Significant clusters for the comparison between PD patients off dopaminergic medication and HC. L, left; R, right; PD, Parkinson's disease; HC, healthy control participants. [Color figure can be viewed at wileyonlinelibrary.com]

We first assessed areas that consistently showed decreased motor‐related activation in PD. Forty‐nine experiments (420 unique subjects; average sample size, 14.0) reported results for the contrast HC > PD. The meta‐analysis revealed significant convergence of activation differences in the left and right posterior putamen (detected in 17 and 18 experiments, respecrively, corresponding to 35% and 37%, respectively, of all experiments), left and right precentral gyrus (12 and 10 experiments, respectively, corresponding to 24% and 20%, respectively), SMA (11 experiments, 22%) and right cerebellar lobule 6 (8 experiments, 16%); see Figure 1A and Table 2. When only considering studies in which PD patients were tested off dopaminergic medication, there were 36 experiments with 345 unique subjects and an average sample size of 14.0 that reported results for the contrast HC > PD‐OFF. Activation differences converged in the left and right posterior putamen (detected in 13 and 14 experiments, respectively, corresponding to 36% and 39%, respectively), left precentral gyrus (8 experiments, 22%), and left cerebellar lobule 5/vermis (7 experiments, 19%); see Figure 1B and Table 2. None of the detected areas showing decreased activation in PD correlated with differences in disease severity across studies, as indexed by mean UPDRS scores (all *P*_uncorrected_ > 0.05; see Methods for more details).

**TABLE 2:** Results of ALE analyses for all between‐group contrasts

	Side	MNI coordinates (at peak)			*Z* value (at peak)
		*x*	*y*	*z*	
Decreased activation in PD compared with HC					
Putamen	Right	30	−10	6	5.78
Putamen	Left	−30	−8	2	6.87
Precentral gyrus	Left	−34	−22	62	7.03
Precentral gyrus	Right	36	−20	72	5.18
Supplementary motor area	Left	−4	−6	58	5.68
Cerebellum, lobule VI	Right	26	−54	−30	4.27
Decreased activation in PD‐OFF compared with HC					
Putamen^a^	Right	30	−10	6	5.26
Putamen	Left	−30	−4	0	6.67
Precentral gyrus	Left	−34	−22	62	5.68
Cerebellum, lobule V/vermis	Left	−6	−60	−14	4.29
Increased activation in PD compared with HC					
Pre‐supplementary motor area	Left	−2	2	58	4.77
Precentral gyrus/middle frontal gyrus	Left	−34	−6	58	4.81
Precentral gyrus/middle frontal gyrus	Right	32	−6	56	4.99
Increased activation in PD‐OFF compared with HC					
Precentral gyrus/middle frontal gyrus	Right	30	−4	56	4.77
Precentral gyrus/middle frontal gyrus	Left	−34	2	52	4.33

Clusters with convergence of activation maxima are reported at a threshold of 0.05 family‐wise error corrected at the cluster level.

^a^ The second peak of the cluster is listed because the first peak was localized in white matter.

### Increased Activation in PD

We then analyzed which areas consistently showed increased motor‐related activation in PD. Forty‐one experiments with 369 unique subjects and an average sample size of 13.9 reported results for the contrast PD > HC. We found significant convergence of activation differences in pre–supplementary motor area (detected in 13 experiments, corresponding to 32%), as well as left and right rostral precentral gyrus/middle frontal gyrus (both detected in 13 experiments, corresponding to 32%); see Figure 1A and Table 2. When limiting the meta‐analysis to studies of PD patients off medication there were 34 experiments with 300 unique subjects and an average sample size of 13.7 that reported results for the contrast PD‐OFF > HC. This meta‐analysis showed significant convergence of activation differences in the left and right rostral precentral gyrus/middle frontal gyrus (detected in 8 and 11 experiments, respectively, corresponding to 24% and 32%, respectively); see Figure 1B and Table 2.

### Probability of Detecting Decreased Putamen Activation in PD

Even though the posterior putamen was the area that was most consistently underactivated in PD, it was only reported in roughly a third of all experiments (see above), which is somewhat surprising given the pivotal role of the putamen in pathophysiological models of PD.^2^ Because we observed that many of the included studies used motor tasks that primarily induced cortical activation, we hypothesized that some of these studies were not suited to detect decreased activation of the putamen in PD because the experimental task or study design was suboptimal for detecting task‐related activity in the putamen. To test this, we analyzed whether a given experiment induced activation of the putamen in the control group and, if so, whether this experiment found abnormal putamen activation in PD. This analysis showed that 21 of the 25 experiments in which putamen activation was found in the healthy control group were able to detect decreased activation of the putamen in PD (corresponding to 84%), whereas only 4 of these experiments (ie, 16%) were not able to detect this difference. There were no experiments that found decreased activation of the putamen in PD without detecting putamen activity in the healthy control group. Thus, when using experimental paradigms that robustly activate the putamen, the probability of detecting hypoactivation in PD is much higher than reflected by the ALE analysis across all tasks (84% vs 35%–39%).

### Meta‐Analytic Connectivity Modeling

**FIG. 2:**
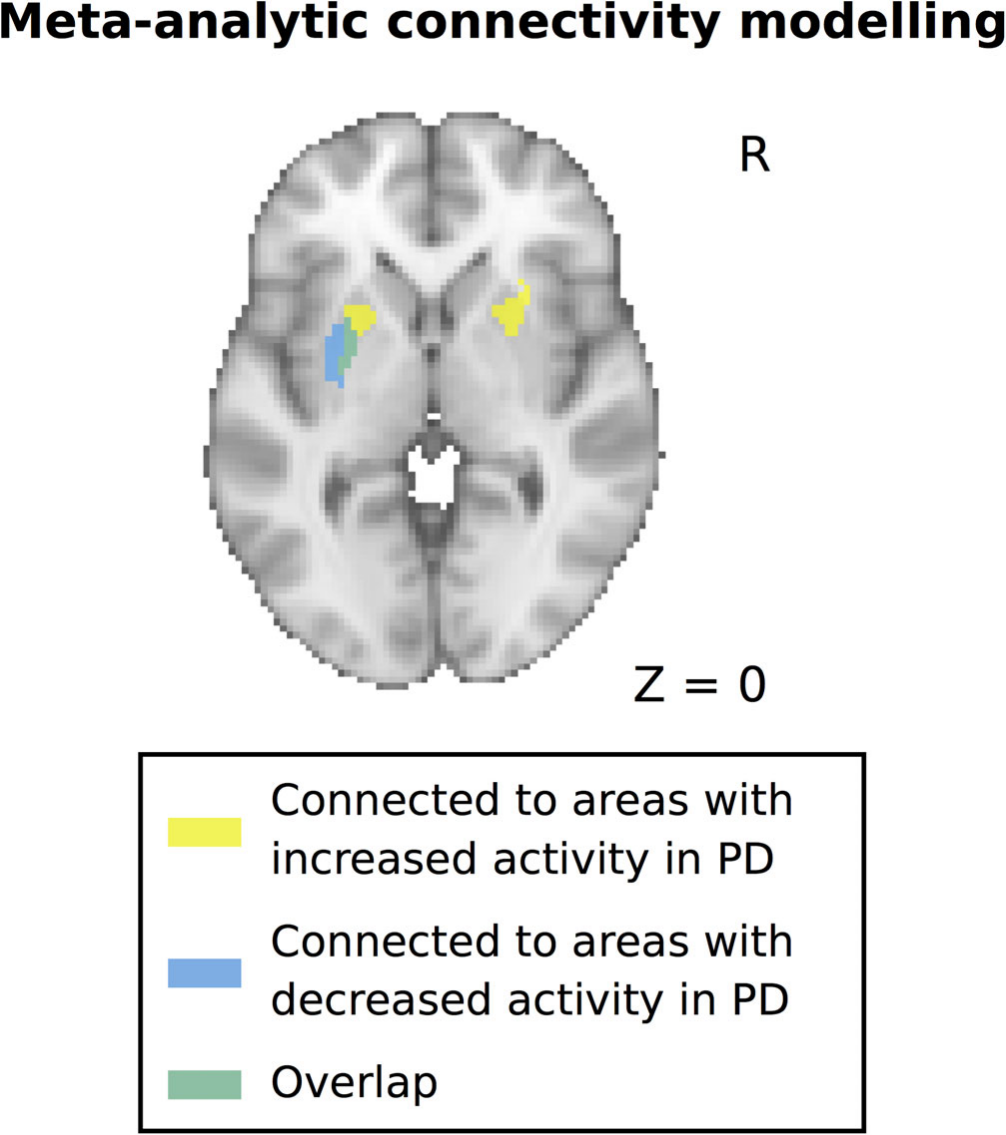
Functional connectivity profiles of areas with decreased and increased activity in PD with the putamen. Functional connectivity was computed using meta‐analytic connectivity modeling and revealed a rostrocaudal gradient for areas with increased versus decreased activity in PD. PD, Parkinson's disease. [Color figure can be viewed at wileyonlinelibrary.com]

Because dopaminergic deafferentation of the putamen in PD shows a prominent caudal‐to‐rostral gradient, we hypothesized that areas showing decreased and increased activation in PD might be connected to distinct subareas of the putamen, with areas showing decreased activity being mainly connected to the more affected caudal (posterior) putamen, which contains the motor territory of the striatum. To test this, we computed functional connectivity profiles of the areas showing abnormal activation in PD using MACM (see Methods for more details). In line with our hypothesis, we found that areas that showed decreased activation in PD were mainly connected with the posterior putamen, whetrsd areas showing increased activation in PD were connected with more anterior parts of the putamen (Fig. 2).

## Discussion

Using a meta‐analytic ALE approach, we found consistent patterns of motor‐related hypo‐ and hyperactivation in several cortical and subcortical areas in PD. The area that most consistently showed decreased activation in PD was the posterior putamen (about 35%–39% of experiments). This finding is in good agreement with previous meta‐analyses^13^, ^58^ as well as single‐photon emission computed tomography and PET studies showing marked dopaminergic denervation of the posterior putamen in PD.^59^ We also found consistent hypoactivation of bilateral M1 and SMA (between 20% and 24% of experiments). Although decreased activation of these areas was less often reported, both areas have long been implicated in the pathophysiology of PD.^19^, ^60^, ^61^ Finally, there was consistent hypoactivation in the cerebellum. Although only relatively few studies reported decreased cerebellar activation (between 16% and 19% of experiments), it should be noted that most of the early studies had a limited field of view, which did not include the cerebellum. Furthermore, several studies reported increased activation of the cerebellum in PD.^62^, ^63^ These discrepancies might be related to differences in the applied motor tasks, different PD phenotypes, or different subareas of the cerebellum. Future meta‐analyses comprising a larger number of studies testing cerebellar activation in PD might help to further clarify the role of altered cerebellar activation in PD. We did not find correlations between reduced activity in these areas and the mean UPDRS scores of the individual studies, suggesting that the observed activity changes do not closely reflect disease progression or, alternatively, that the group average UPDRS scores are not sensitive enough for elucidating this relationship.

Most included studies did not report activation changes in all, but only in a subset of these areas, and a common underactivation only became evident in this meta‐analytic approach. However, there is evidence from multivariate analyses of neuroimaging data that PD is related to a network dysfunction rather than abnormal function of isolated neural areas.^64^ Overlaying meta‐analytic functional connectivity maps of the hypoactivated cortical areas on an anatomical map of the putamen revealed that these areas share a common pathway through the posterior putamen, the striatal area that is most affected by dopaminergic denervation in PD.^65^ Dopaminergic denervation is thought to result in an imbalance between a net inhibitory (indirect) and net facilitatory (direct) pathway that connects the cortex with the basal ganglia in a closed‐loop fashion.^66^ This results in abnormal inhibition of the cortex by the basal ganglia that can be further modified by the cerebellum, which shares reciprocal disynaptic connections with the basal ganglia.^67^ The loop running through the posterior putamen is often referred to as a “motor loop” because it is thought to be primarily involved in processes related to movement execution and habitual movements.^66^

Does the reduced task‐related activation of this network in PD have a correlate at the behavioral level? Although reverse inference should be taken with caution,^68^ there is strong evidence from neuroimaging and electrophysiological studies for a critical role of this network in the modulation of movement vigor. This has been demonstrated for the posterior putamen,^69^, ^70^, ^71^, ^72^ SMA,^70^, ^73^ M1,^72^, ^74^, ^75^, ^76^, ^77^ and the cerebellum.^71^, ^72^, ^77^, ^78^, ^79^ Furthermore, it should be noted that this meta‐analysis was conducted in studies using a variety of motor tasks implying that any detected difference should not be specific to a certain kind of movement, but rather a general process underlying motor execution. We speculate that the process that is probed in many of these neuroimaging studies in PD might be the modulation of movement vigor, which is a crucial aspect of motor control,^80^ and reduced movement vigor constitutes a core motor impairment in PD (clinically termed bradykinesia). This idea is supported by several neuroimaging studies in PD that directly tested movement vigor, for example, by recording force production, and found decreased activation in the posterior putamen, precentral gyrus, SMA, and cerebellum.^5^, ^7^, ^36^, ^79^

We also detected areas that consistently showed increased activation in PD, a midline cluster primarily involving the pre‐SMA (32% of experiments) and the bilateral rostral precentral gyrus/middle frontal gyrus (24%–32% of experiments). Interestingly, both the midline cluster and the more lateral clusters were localized directly anterior to areas that showed decreased activation in PD, namely, SMA and bilateral precentral gyrus (see Fig. 1). Anatomical studies have demonstrated a rostrocaudal gradient in both the medial prefrontal cortex (comprising the pre‐SMA and SMA) and the premotor cortex, where the more rostral areas are connected to the prefrontal areas, whereas the more caudal areas are connected to the primary motor cortex and the spinal cord.^81^ This gradient is also reflected in distinct connectivity patterns with the basal ganglia, where more rostral cortical areas are connected to more rostral (and ventral) parts of the striatum.^82^ The more rostrally localized loop is often referred to as the “associative” loop and is thought to be primarily related to executive control of movements and goal‐directed behavior.^66^, ^83^ In line with these previous studies, the meta‐analytic functional connectivity profiles of pre‐SMA and premotor cortex in the MACM analysis showed common coactivation with the anterior putamen, which is relatively spared from dopaminergic denervation in PD. Of note, this coactivation was observed bilaterally, which might indicate less lateralization of this loop compared with the motor loop running through the posterior putamen. It has previously been suggested that PD patients might rely more on effortful or “goal‐directed” behavior, which is related to the associative cortical–basal ganglia loop, because more “automatic” motor behavior, which has been related to the motor cortical–basal ganglia loop, is impaired.^84^ Similarly, it has been suggested that PD patients recruit areas that are involved in externally cued movements to compensate for impairments in internally generated movements.^85^ However, this remains speculative, and it should be noted that increased cortical activation of rostral motor areas in PD might not exclusively have compensatory effects but could also have deleterious effects. For example, increased activation of the pre‐SMA in PD has been demonstrated in patients developing involuntary “dyskinesia” movements as a side effect of dopaminergic therapy.^86^, ^87^ Elucidating the role of these areas in PD warrants further research.

In conclusion, we were able to detect distinct neural networks showing decreased and increased motor‐related activation in PD using a meta‐analytic approach. Meta‐analyses should be continuously updated because the increasing number of studies that can be included further increases the sample size and reduces ambiguity of the results (see, eg, the current meta‐analysis and our previous analysis from 2014). This might also allow analyzing contrasts that we were not able to test in the current analysis because of the limited number of individual experiments, such as PD‐ON versus PD‐OFF to elucidate effects of dopaminergic medication on neural activity in PD. To facilitate this, we will make all data from this meta‐analysis publicly available on ANIMA (anima.inm7.de), including Excel sheets with the coordinates from all studies, the ALE software, and corresponding scripts. This allows replication of the results and will hopefully facilitate revised meta‐analyses in the future.

## Author Roles

D.M.H.: research project conception, organization, execution; statistical analysis design, execution; manuscript writing of the first draft.

D.M.: research project execution. Statistical analysis execution; manuscript review and critique.

J.C.: statistical analysis design, execution; manuscript review and critique.

S.B.E.: research project conception, organization; statistical analysis review and critique; manuscript review and critique.

H.R.S.: research project conception, organization; statistical analysis review and critique; manuscript review and critique.

## Financial Disclosures

D.M.H., D.M., J.A.C., and S.B.E. have nothing to declare. H.R.S. has received honoraria as speaker from Sanofi Genzyme, Denmark, and Novartis, Denmark, as consultant from Sanofi Genzyme, Denmark and Lundbeck AS, Denmark, as editor‐in‐chief (*Neuroimage Clinical*) and senior editor (*NeuroImage*) from Elsevier Publishers, Amsterdam, The Netherlands. He has received royalties as book editor from Springer Publishers, Stuttgart, Germany, and from Gyldendal Publishers, Copenhagen, Denmark.
